# Exercise training improves vascular function in patients with Alzheimer’s disease

**DOI:** 10.1007/s00421-020-04447-w

**Published:** 2020-07-30

**Authors:** Anna Pedrinolla, Massimo Venturelli, Cristina Fonte, Stefano Tamburin, Angela Di Baldassarre, Fabio Naro, Valentina Varalta, Gaia Giuriato, Barbara Ghinassi, Ettore Muti, Nicola Smania, Federico Schena

**Affiliations:** 1 Department of Neurosciences, Biomedicine and Movement Sciences, University of Verona, Via Casorati 43, 37131 Verona, Italy; 2 Department of Internal Medicine, University of Utah, Salt Lake, Utah USA; 3 Department of Neurosciences, Biomedicine and Movement Sciences, Neuromotor and Cognitive Rehabilitation Research Centre, University of Verona, Verona, Italy; 4 Department of Medicine and Aging Sciences, University G. D’Annunzio, Chieti-Pescara, Chieti, Italy; 5 Department of Anatomical, Histological, Forensic Medicine and Orthopedic Science, Rome, Italy; 6 Mons. A. Mazzali Foundation, Mantua, Italy

**Keywords:** Dementia, Physical activity, Flow-mediated dilation, Passive-leg movement, Vascular endothelial growth factor

## Abstract

**Purpose:**

Vascular dysfunction has been demonstrated in patients with Alzheimer’s disease (AD). Exercise is known to positively affect vascular function. Thus, the aim of our study was to investigate exercise-induced effects on vascular function in AD.

**Methods:**

Thirty-nine patients with AD (79 ± 8 years) were recruited and randomly assigned to exercise training (EX, *n* = 20) or control group (CTRL, *n* = 19). All subjects performed 72 treatment sessions (90 min, 3 t/w). EX included moderate–high-intensity aerobic and strength training. CTRL included cognitive stimuli (visual, verbal, auditive). Before and after the 6-month treatment, the vascular function was measured by passive-leg movement test (PLM, calculating the variation in blood flow: ∆peak; and area under the curve: AUC) tests, and flow-mediated dilation (FMD, %). A blood sample was analyzed for vascular endothelial growth factor (VEGF). Arterial blood flow (BF) and shear rate (SR) were measured during EX and CTRL during a typical treatment session.

**Results:**

EX group has increased FMD% (+ 3.725%, *p* < 0.001), PLM ∆peak (+ 99.056 ml/min, *p* = 0.004), AUC (+ 37.359AU, *p* = 0.037) and VEGF (+ 8.825 pg/ml, *p* = 0.004). In the CTRL group, no difference between pre- and post-treatment was found for any variable. Increase in BF and SR was demonstrated during EX (BF + 123%, *p* < 0.05; SR + 134%, *p* < 0.05), but not during CTRL treatment.

**Conclusion:**

Exercise training improves peripheral vascular function in AD. These ameliorations may be due to the repetitive increase in SR during exercise which triggers NO and VEGF upregulation. This approach might be included in standard AD clinical practice as an effective strategy to treat vascular dysfunction in this population.

## Introduction

Alzheimer’s disease (AD) is one of the most common age-related diseases (Alzheimer’s Association 2019), mainly known for its cognitive symptoms that interfere with activities of daily life and impact memory loss, learning, language, and behavior (Alzheimer’s Association 2019; Rhodin and Thomas 2001). AD is degenerative, it probably begins several years before symptoms arise and individuals experience noticeable cognitive impairment only after years of brain changes (Alzheimer’s Association 2019; Rhodin and Thomas 2001). Neurons of specific areas of the brain undergo degeneration in AD due to the accumulation of the protein fragment beta-amyloid (Aβ plaques) outside neurons and the accumulation of an abnormal form of the tau protein (neurofibrillary tangles) (Alzheimer’s Association 2019). Despite decades of investigations focused on understanding the mechanisms that cause these changes in the brain in AD, to date, there is no treatment for AD, and also no interventions to delay or prevent AD, or to alleviate symptoms and comorbidities (Dede et al. 2007; Barnes and Corkery 2018; Rhodin and Thomas 2001).

Interestingly, several recent studies explored mechanisms different from Aβ plaque deposition and neurofibrillary tangles that may be involved in AD onset and development (Rhodin and Thomas 2001; Barnes and Corkery 2018; Eldholm et al. 2018; Sweeney et al. 2019; Pedrinolla et al. 2017; Venturelli et al. 2018). There is increasing evidence regarding vascular changes in AD (Dede et al. 2007; Sweeney et al. 2019). Cerebral capillary atrophy, focal constriction, reduced perfusion of the temporal and frontal cortices, structural changes in endothelial cells, Aβ deposition in vessel walls are some of the vascular changes that have been reported in individuals with AD. Based on these findings, it has been hypothesized that the primary site of the onset of AD, and the target of the toxic Aβ are vessels, small arteries, arterioles, and capillaries of the central nervous system (Rhodin and Thomas 2001; Dede et al. 2007; Venturelli et al. 2018). Vascular dysfunction has also been documented at systemic level in individuals with AD. Specifically, a reduced bioavailability of nitric oxide (NO) accompanied by a reduction in blood flow inward from extracranial conduit arteries, independently from vascular risk factors, has been reported (Dede et al. 2007; Venturelli et al. 2018; Sweeney et al. 2019; Grammas et al. 2014; Popa-Wagner et al. 2015). Cerebral, extracranial and peripheral vascular dysfunction appeared to have a linear relationship with cognitive dysfunction (Venturelli et al. 2018) suggesting that vascular function (both central and peripheral) may play a key role in AD.

The robust evidence supporting the contribution of vascular dysfunction in AD development offers the opportunity of targeting these factors to prevent, delay or reverse further progression and inherent cognitive deterioration (Picano et al. 2014; Alagiakrishnan et al. 2006). It is well known that exercise training has a strong impact on vascular function, and it is known to be one of the most potent non-pharmacological vaso-protective interventions (Picano et al. 2014; Pierce et al. 2011). Indeed, it is well established that exercise training has a direct effect on blood flow (BF), vasoreactivity, vascular growth factors (i.e., vascular endothelial growth factor, VEGF), and angiogenesis at systemic level and all those responses are mediated by the up-regulation of NO-bioavailability and growth factor expression (Mann and Rosenzweig 2012; Izzicupo et al. 2017). Although the exercise-induced benefits on vascular and cognitive function in AD are increasingly appreciated (Barnes 2015; Najar et al. 2019; Alagiakrishnan et al. 2006; Pedrinolla et al. 2018; Fonte et al. 2019), the mechanisms by which exercise training may induce positive peripheral vascular adaptations in individuals with AD are still matter of debate, and have not been studied in human models.

Therefore, the aim of this study was to investigate the effect of exercise training (EX) on systemic vascular function of individuals with AD, and examine the mechanisms underlying this non-pharmacological intervention. Our main hypothesis was that exercise training induces positive peripheral vascular adaptations likely triggered by the great increase in BF and shear rate (SR) during exercise training that would serve as a stimulus for induce metabolites production and upregulation of growth factors.

## Methods

### Study design

A single-blind randomized controlled trial (RCT) comparing the effects of EX with control group (CTRL) on systemic vascular function and VEGF. The examiners were blinded to group assignment (allocation ratio 1:1). The study was reported in accordance with the CONSORT guidelines.

### Participants

Individuals with AD were recruited from the Department of Neurosciences, Biomedicine and Movement Sciences, University of Verona, Italy, and Mons. Mazzali Geriatric Institute, Mantua, Italy, between January 2014 and February 2016. Inclusion criteria were (1) age between 65 and 90 years; (2) clinical diagnosis of probable AD dementia, established according to the National Institute on Aging-Alzheimer’s Association diagnostic guideline for AD (McKhann et al. 2011b); (3) Performance Oriented Mobility Assessment (POMA) score > 19 (McKhann et al. 2011a; Albert et al. 2011). Exclusion criteria were (1) modifications of medications during the last 3 months, (2) history of depression or psychosis, alcohol or drug abuse, other neurological, cardiac, orthopedic, or respiratory pathology (e.g., chronic obstructive pulmonary disease); (3) Mini-Mental State Examination (MMSE) score < 10 and Clinical Dementia Rating (CDR) Scale score = 0. At the time of the inclusion, MMSE was performed to evaluate the severity of the disease.

**Fig. 1:**
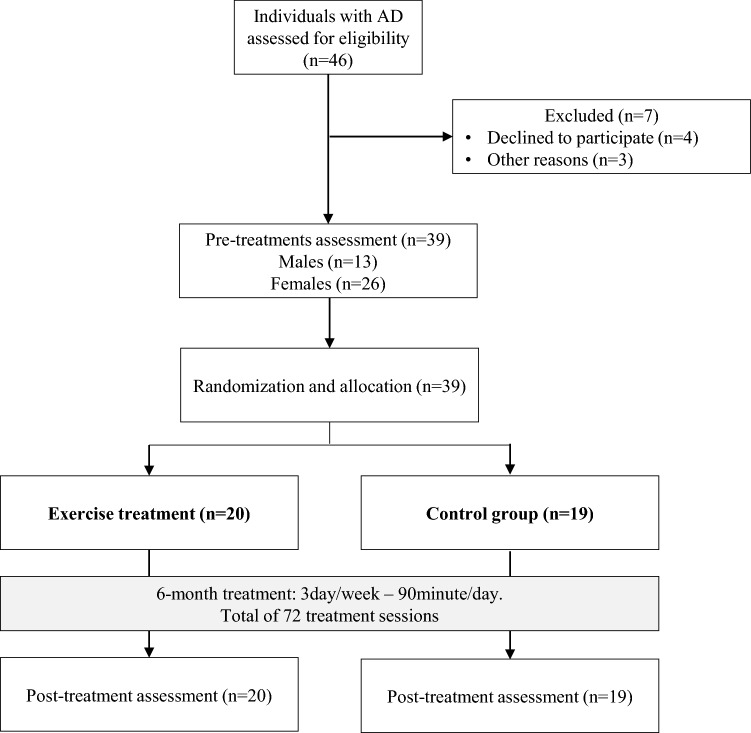
Flow diagram of the randomized controlled trial

At the first visit, patients, or their relatives on behalf of them, declared their experience with exercise. Most of them have practiced exclusively light aerobic exercise in the past, while none of them had experience with strength exercise. After the first evaluation, patients were randomly assigned to EX or CTRL. During the study, all patients maintained their regular pharmacological therapy, and if there were some changes, they had to communicate it to the responsible of the study. The flowchart of the study is reported in Fig. 1. Patients and their relatives were informed about the experimental nature of the study and gave their written informed consent.

### Interventions

All individuals included in the study took part in a total of 72 treatment sessions, 90-min/day, 3 days/week. Both treatments lasted 6 months (± 15 days). The compliance to the treatment was 100% since when a participant cold not take part in a session for any reason, there was always the chance to recover the missed session. The treatment sessions consisted of group activities and included seven–eight participants. During the study, patients were asked not to attend other types of exercise trainings.

#### Exercise training (EX)

EX was conducted by two kinesiologists (ratio 2:5), included moderate intensity endurance and resistance training. Sessions started with 15 min of warm-up which included active joint mobilization. Then patients underwent a total of 45 min of endurance exercises divided into 15 min of cycling on cycle ergometer, 15 min of walking on a treadmill, and 15 min of arm cranking on a specific ergometer with a random order. The 70% of the maximal heart rate was calculated using the Karvonen formula (i.e., 220 − age in years) (Karvonen and Vuorimaa 1988) because no specific equation is validated in patients with dementia. For participants taking beta-blockers, the 65% of (220 − age) was considered, as suggested by Carvalho et al. (2008). For each endurance exercise, workload intensity was increased, if possible, by 5% every 4 weeks, based on the individuals’ heart rate (HR), as measured via a HR monitor. The RPE scale was used as a tool to monitor how patients perceived the effort during the aerobic training session (Yu et al. 2015). Although participants were not completely naïf to aerobic exercise, exercising at a certain intensity for some minutes was not easy for them, especially at the beginning of the study. The RPE scale was useful to monitor the global effort experienced by the patients during the training. Furthermore, patients started with a low-intensity endurance training in the first EX sessions, aiming to reach the 70% intensity in 2–3 weeks. This allowed to set the right intensity for all the exercises included in this training session, in particular for the arm cracking device which may be more challenging than other training equipment. As expected, all the participants reached the required intensity within 2–3 weeks.

After endurance exercises, patients performed resistance exercise with 3 sets of 12 reps at 85% of 1 repetition maximum (1RM), estimated with the Brzicky method, for isotonic ergometers including chest-press, leg-curl, leg-extension, lat-machine, and leg-press. Selected patients were all naïf to resistance training and due to the short familiarization (1 day) with exercise devices, the 1RM was likely underestimated. Therefore, during the first week of EX, we asked the participants to perform as many repetitions as possible with the 85% of the estimated 1RM. Furthermore, as soon as participants were able to perform the 12 repetitions easily (i.e., they were able to execute more than 12 repetitions), the workload was increased by 5%. EX ended with stretching exercises for all the muscles involved in the training. The kinesiologists motivated the participants and gave patients time to perform the exercise as a whole.

#### Control group (CTRL)

Patients with AD included in CTRL group performed regular cognitive therapy. The standard therapy was conducted by two neuropsychologists (ratio 2:5) and did not include any kind of movement. Indeed, during the sessions, participants were sitting around a table where they performed cognitive tasks including 90 min of multi-modal stimuli (visual, verbal, auditive, tactile) to stimulate residual abilities of the patients, in particular attention and memory functions, using sensorial material and repeated short-term tasks (Fonte et al. 2019).

### Outcome measures

Outcome measures were assessed by the same blinded examiners before (pre) and immediately after (post) 6 months of treatment. All subjects came to the lab in the morning, between 9.00 and 10.00 AM in a fasted state and in abstinence from alcohol, caffeine, and physical activity for at least 12 h (Harris et al. 2010). First, a blood sample was collected, and then vascular function assessment was carried out, taking approximately 40 min. Consequently, two functional tests, 6-min walking test (6-MWT) and physical-performance test (PPT) were performed, taking about 20 min with a short rest between tests.

#### Passive limb movement test (PLM)

Recent investigations suggested that PLM-induced hyperemia is predominantly a consequence of NO-mediated vasodilation (Trinity et al. 2012; Venturelli et al. 2018). Therefore, we have adopted this noninvasive and reliable method to determine NO bioavailability Measurements of femoral arterial blood velocity and vessel diameter were performed in the passively moved leg distal to the inguinal ligament and proximal to the deep and superficial femoral bifurcation with a Doppler ultrasound system (GE Logiq-7). The ultrasound system was equipped with a linear transducer operating at an imaging frequency of 10 MHz. Vessel diameter was determined at a perpendicular angle along the central axis of the scanned areas. Blood velocity was measured using the same transuducer with a frequency of 5 Hz. All blood velocity measurements were obtained with the probe appropriately positioned to maintain an insonation angle of 60° or less. The sample volume was maximized according to vessel size and was centered within the vessel. Arterial diameter was measured and mean velocity was automatically calculated by the Doppler ultrasound. With the use of femoral arterial diameter and blood velocity (*V*_mean_), femoral blood flow (BF) was calculated as

$${\text{BF }} = \, V_{{{\text{mean}}}} \cdot\Pi \left( {{\text{vessel diameter}}/{2}} \right)^{{2}} {6}0.$$

The PLM protocol consisted of 60 s of resting baseline femoral blood flow data collection, followed by 60 s of passive knee extension and flexion with the same measure. PLM was performed by a member of the research team, who moved the subject’s lower leg through a 90° range of motion (180–90° knee joint angle) at 1 Hz. Blood *V*_mean_ was analyzed with 1 Hz resolution on the Doppler ultrasound system for 60 s at rest and second by second for the first 60 s following the initiation of PLM. Peak blood flow relative changes (BF ∆peak) from rest, and the area under the curve (AUC) of femoral blood flow was determined for each subject (Venturelli et al. 2018).

#### Flow-mediated dilation (FMD)

FMD test represents a functional bioassay for endothelium-derived nitric oxide (NO) bioavailability and vascular function in humans (Harris et al. 2010). The FMD test was performed in a quiet room, and during this evaluation, subjects rested in the upright-seated position for 20 min before the start of data collection and remained in this position throughout this part of the study. Briefly, high-resolution ultrasound was used to image the brachial artery at rest and after 5 min of ischemia. All the FMD were performed with the participant in the supine position, with the right arm extended at an angle of ~ 90° from the torso. The brachial artery was imaged using a high-resolution Logiq-7 ultrasound Doppler system equipped with a 12–14 MHz linear array transducer (General Electric Medical Systems, Milwaukee, WI, USA). The brachial artery was imaged 5–10 cm above the antecubital fossa in the longitudinal plan, and the diameter was determined at 90° angle along the central axis of the scanned area. When an optimal image was acquired, the position was maintained for the whole test and all scans were stored for later analysis. After baseline brachial artery imaging (basal measurement), a blood pressure cuff was placed around the forearm and inflated to 250 mm Hg for 5 min. Brachial artery images and blood velocity were obtained continuously 30 s before and 2 min after cuff release (Harris et al. 2010). The brachial artery images were analyzed by a blinded investigator by means of FloWave.US (Coolbaugh et al. 2016). Arterial diameter was measured as the distance (mm) between the intima–lumen interfaces for the anterior and posterior walls.

FMD was calculated as a percentage change of the peak diameter in response to reactive hyperemia in relation to the baseline diameter, according to the following equation:

$${\text{FMD }}\left( \% \right) \, = \, \left( {{\text{peak diameter}} - {\text{baseline diameter}}} \right)/{\text{baseline diameter}},$$

and, when multiplied by 100, FMD was expressed as a percentage of change in the vessel caliber (Harris et al. 2010).

#### Blood sample and vascular endothelial growth factor (VEGF)

Plasma was obtained from 12-h fasting venous blood samples and stored at – 80 °C until analysis. Plasma VEGF concentration was determined with an enzyme-linked immunosorbent assay (DRG International Inc., Mountainside, NJ, USA) according to the manufacturer’s suggestions. All samples were analyzed in duplicate during the same assay session.

#### Blood flow and shear rate during treatments

BF and SR at brachial and common femoral arteries were measured in each participant during one session of EX and one session of CTRL about halfway of the treatment period (after about 3 months). During EX, brachial and common femoral arteries were scanned at baseline (record 0) and after each endurance (records 1, 2, and 3) and resistance exercise (records 4, and 5) bout (about every 15 min). During CTRL, the same timing used for EX was adopted, and arteries were imaged using the same high-resolution Logiq-7 ultrasound Doppler system equipped with a 12–14 MHz linear array transducer. First, a basal record of 60 s was taken right before the starting of the treatment. The arteries images were analyzed by a blinded investigator by means of FloWave.US (Coolbaugh et al. 2016). Arterial diameter was measured as the distance (mm) between the intima–lumen interfaces for the anterior and posterior walls. BF and SR were calculated using arterial diameter blood velocity according to these formulae (Harris et al. 2010):

- $${\text{BF }}\left( {{\text{ml}}/{\min}} \right) \, = {\text{ blood velocity }}* \, \pi * \, \left( {{\text{vessel diameter}}/{2}} \right)^{{2}} *{ 6}0,$$
- $${\text{SR }}\left( {s^{{ - {1}}} } \right) \, = { 8}V_{{{\text{mean}}}} /{\text{vessel diameter}}$$.

#### Six-minute walking test (6-MWT)

The 6-MWT measures the maximum distance that a person can walk over 6 min and it is commonly used as an assessment of exercise capacity. The participants were instructed to walk from one end of a 15-m course to the other and back again as many times as possible in 6 min, while under the supervision of a kinesiologist. After each minute, participants were informed of the time elapsed and were given standardized encouragement. The distance (meters) covered in 6 min was recorded (Makizako et al. 2013).

#### Physical performance test (PPT)

The nine-item PPT assesses physical function competences. The following maneuvers simulating daily activities were assessed: writing a sentence, simulation of eating, rising up and putting a heavy book in a shelf, dressing and taking off a jacket, picking up a coin from the floor, turning 360°, gait test, climbing stairs, number of flights during climbing the stairs. Seven of the nine tasks were timed and the scores for time intervals of each task were given, from 0 if task was unable to be performed to 4 if it was performed at its possible best. During the 360° turn, stability and continuity of turning were assessed. The maximum score for the nine items is 36 points (Stożek et al. 2016).

### Randomization and masking

After screening, participants were allocated to one of the two arms according to a simple software-generated randomization scheme (www.randomization.com): (1) EX group, and (2) CTRL group. The research team included “evaluators” and “treatment givers”. Evaluators were uninformed about group assignments, including physician and neuropsychologist who performed outcome measures. Treatment givers included neuropsychologists and kinesiologists who administered EX and CTRL, respectively.

### Statistical analysis

All statistical analyses were performed with Sigma PLOT Windows Version 14.0 (Systat Software, Chicago, IL). Data are presented as mean ± SD. First, normality was assessed by the Shapiro–Wilk test. A one-way (1 × 2) analysis of variance (ANOVA) was applied to age, education, MMSE, POMA, CDR, height (m), and weight (kg) between groups to test the homogeneity of the groups before the study. A two-way (2 × 2) ANOVA, with “Time” as within-group factor, and “Treatment” (EX and CTRL) as between-group factors was applied to primary and secondary outcomes. A two-way (2 × 6) rm-ANOVA, with “Time” as a within-group factor (records 0, 1, 2, 3, 4, 5) and “Treatment” (EX and CTRL) as between-group factor, was applied to BF and SR.

In the presence of significant effects, a multiple comparisons test with Bonferroni’s correction was performed. The family-wise alpha level for significance was set at 0.05 (two tails), with Bonferroni’s correction when needed, for all the analyses.

## Results

### Demographic and clinical baseline data

The flow diagram of the study with the specific numbers of participants is reported in Fig. 1. Thirty-nine individuals with AD (13 males and 26 females) were included in the study and randomly assigned to EX (*n* = 20) and CTRL groups (*n* = 19).

Age, education, MMSE and POMA were not statistically different between the two groups at baseline. Demographic and clinical characteristics of the patients are reported in Table 1. Primary and secondary outcomes measures did not significantly differ between the two groups at baseline (Table 2).

**Table 1:** Subjects’ characteristics, pharmacological treatment, and comorbidities

	EX	CTRL
Number	20	19
Male/Female—*n*	6/14	7/12
Age—years	79 ± 7	79 ± 9
Education—years	7 ± 4	8 ± 5
MMSE—(0–30)	17.8 ± 5.7	19.6 ± 4.3
ADAS-Cog—(0–70)	30.4 ± 16.9	26.8 ± 7.5
POMA—(0–28)	22.7 ± 2.9	22.9 ± 3.7
CDR—(0–3)	*n*9 = 1; *n*11 = 2	*n*9 = 1; *n*10 = 2
Height—cm	162	165
Weight—kg	67.4	65.4
Pharmacological treatment		
Cholinesterase inhibitors—*n*	9	9
Antipsychotics—*n*	5	4
Benzodiazepines—*n*	1	2
Comorbidities		
Hypertension—*n*	8	13
Diabetes—*n*	1	1
Arthrosis—*n*	4	1

Data are given as mean ± standard deviation

*EX* exercise treatment group, *CTRL* control group, *MMSE* Mini-Mental State Examination, *ADAS-Cog* Cognitive section of the Alzheimer’s disease Assessment Scale, *POMA* Performance Oriented Mobility Assessment, *CDR* Clinical Dementia Rating Scale

**Table 2:** Outcome's baseline values

	EX	CTRL
Number	20	19
FMD—%	9.7 ± 4.1	8.6 ± 3.9
BFpeak—ml/min	573 ± 248	601 ± 239
BF ∆peak—ml/min	215 ± 86	271 ± 142
BF AUC—ml/min	57 ± 45	95 ± 84
VEGF—pg/ml	24.8 ± 8.1	29.7 ± 10.2
6-MWT—m	342 ± 53	329 ± 61
PPT—n	18.5 ± 2.5	18.0 ± 2.5

Data are given as mean ± standard deviation

*EX* exercise treatment group, *CTRL* control group, *FMD* flow-mediated dilation, *BF* blood flow, *AUC* area under the curve, *VEGF* vascular endothelial growth factor, *6-MWT* six-minute walking test, *PPT* physical performance test

### Outcome measures

**Fig. 2:**
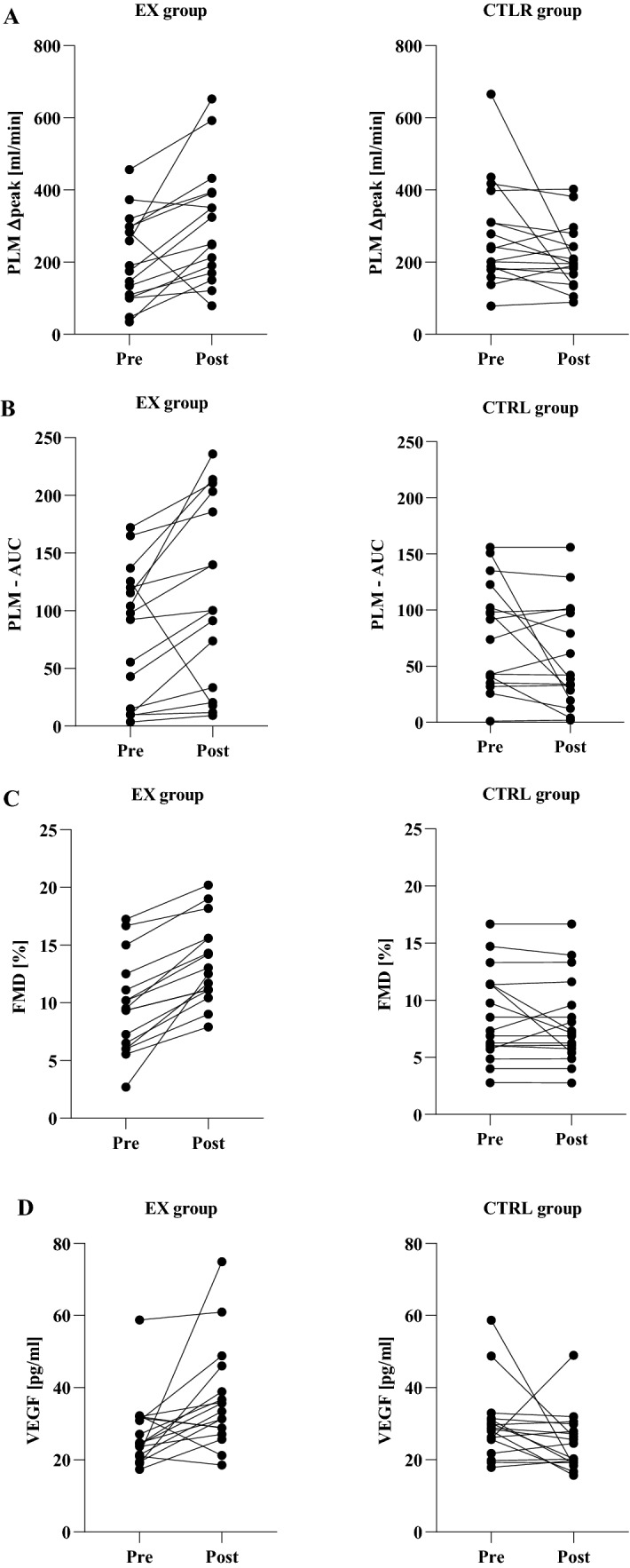
Vascular outcomes. Data are given as mean ± standard deviation. Pre- and post-treatment values for passive limb movement test (PLM): blood flow delta peak (**a**) and area under the curve (AUC, **b**), and flow-mediated dilation (FMD, **c**), and as well as vascular endothelial growth factor (VEGF, **d**). ^†^Within group difference with *p* < 0.05; ^‡^between groups difference with *p* < 0.05. *Ctrl* control group, *EX* exercise treatment

Within-group difference was found in PLM ∆peak only in EX group (within-group mean difference: 99.056 ml·min^−1^, *p* = 0.004). Post-treatment between-group difference was found (between-group mean difference: 91.429 ml·min^−1^, *p* = 0.005; Fig. 2a).

Within-group difference was found in PLM AUC only in EX group (within-group mean difference: 37.359 ml·min^−1^, *p* = 0.037). Post-treatment between-group difference was also detected (between-group mean difference: 54.019 ml·min^−1^, *p* = 0.029; Fig. 2b).

### FMD

Within-group difference was found in FMD % in EX group (within-group mean difference: 3.725%, p  ≤ 0.001) but not in CTRL group. Post-treatment between-group difference was found for this variable (between-group mean difference: 5.296%, *p* < 0.001; Fig. 2c).

### VEGF

Within-group difference was found in VEGF in EX group (within-group mean difference: 8.825 pg·ml^−1^, *p* = 0.004) but not in CTRL group. Post-treatment between groups difference was also detected (between-group mean difference: 10.728 pg·ml^−1^, *p* = 0.011; Fig. 2d).

#### Blood flow and shear rate during treatment

**Fig. 3:**
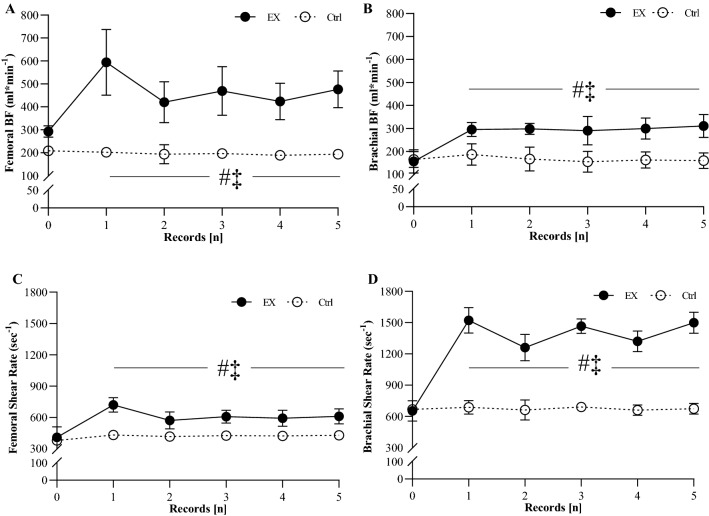
Vascular response during treatments. Values of femoral (**a**) and brachial (**b**) blood flow (BF); femoral (**c**) and brachial (**d**) shear rate during EX (closed circle) and Ctrl (opened circle). Vascular measures were recorded at half-way of the treatments (about 3 months after the starting of the treatments). During EX, measures were taken at baseline and after each bout of aerobic and strength exercise (about every 15 min). Thus, record 0 = baseline, 1–2–3: end of endurance exercise, records 4–5: end of resistance exercise. The same timing was used for measurements during Ctrl. ^‡^significantly different from SE, *p* < 0.05; ^#^significantly different from baseline values, *p* < 0.05

Regarding BF and SR measured at brachial and femoral arteries during EX and CTRL, no between-group differences were detected for baseline values (Fig. 3). Tables 3 shows variation from baseline of BF and SR in EX and CTRL. Within-group differences were found for each time point (1–5) with baseline values (0) in EX group for both BF and SR at femoral and brachial arteries. CTRL group did not show any within-group difference. Between-group differences were found for both BF and SR at points 1–5 (Table 3; Fig. 3a–d).

**Table 3:** Blood flow and Shear rate change during a typical treatment session

Time points	Blood flow [ml*min^−1^]								Shear rate [s^−1^]							
	EX				CTRL				EX				CTRL			
	Femoral artery		Brachial artery		Femoral artery		Brachial artery		Femoral artery		Brachial artery		Femoral artery		Brachial artery	
	∆	*p* value	∆	*p* value	∆	*p* value	∆	*p* value	∆	*p* value	∆	*p* value	∆	*p* value	∆	*p* value
1	317 ± 143	0.023	158 ± 23	0.039	0 ± 10	0.987	20 ± 30	0.991	300 ± 50	0.029	932 ± 138	< 0.001	10 ± 2	0.976	4 ± 20	0.984
2	139 ± 92	0.031	156 ± 15	0.032	2 ± 35	0.923	12 ± 32	0.998	304 ± 52	0.048	673 ± 129	0.003	9 ± 1	0.984	5 ± 43	0.983
3	165 ± 102	0.029	151 ± 45	0.041	3 ± 8	0.998	2 ± 23	0.979	276 ± 32	0.038	874 ± 76	< 0.001	5 ± 3	0.999	4 ± 10	0.998
4	148 ± 98	0.033	163 ± 31	0.031	1 ± 3	0.999	2 ± 14	0.988	287 ± 29	0.033	786 ± 105	0.002	7 ± 3	0.999	4 ± 15	0.995
5	198 ± 87	0.029	165 ± 29	0.041	0 ± 2	0.999	1 ± 12	0.992	279 ± 31	0.038	912 ± 115	< 0.001	10 ± 2	0.999	5 ± 5	0.998

Data are given as mean ± standard deviation. During EX, measures were taken at baseline and after each bout of aerobic and strength exercise (about every 15 min). Table shows variation from the baseline values of blood flow and shear rate recorded at femoral and brachial artery. 1–3–5 time points = end of aerobic exercise, records 2–4 time points: end of strength exercise. The same timing was used for measurements during CTRL (one record every 15 min)

*EX* exercise group, *CTRL* control group

#### Functional tests

Regarding the 6-MWT and PPT, no baseline between-group differences were found (Table 2). Within-group difference was found in 6-MWT in EX group (within-group mean difference: 70.5 m, *p* = 0.002) but not in CTRL group. Post-treatment between-group difference was also detected (between-group mean difference: 91.3 m, *p* = 0.001). The same trend was exhibited for the PPT where within-group difference was found in EX group (within-group mean difference: 2.5 points, *p* = 0.023) but not in CTRL group. Post-treatment between-group difference was also detected (between-group mean difference: 2.0 points, *p* = 0.039).

#### Exercise workload in the EX group

The workload of each training was monitored and modified to keep the right training intensity. Every 4 weeks if possible, the workload for both aerobic and resistance exercises was increased by 5%. Table 4 shows the change between pre- and post-training of the workload for each exercise included in the training session. Averagely, the workload of the full session after 6 months of training significantly increased by 26%.

**Table 4:** Pre- and post-training workload change in EX group

	Pre	Post	∆%	*p*
Aerobic exercises				
Cycle ergometer—W	75 ± 15	95 ± 17	28	0.034
Treadmill—km/h	3.5 ± 0.9	4.6 ± 0.5	32	0.002
Arm cracking—W	15 ± 5	19 ± 7	25	0.028
Resistance exercises				
Chest press—kg	15 ± 3	19 ± 2	25	0.006
Leg curl—kg	18 ± 5	22 ± 2.5	20	0.035
Leg extension—kg	20 ± 5	25 ± 3	25	0.041
Lat machine—kg	20 ± 2	24 ± 2	20	0.004
Leg press—kg	35 ± 5	48 ± 4	37	0.002

Workload for aerobic exercises corresponds to W and km/h kept by subjects for the 15-min exercise. Workload for resistance exercises corresponds to the weight used to perform 3 sets of 12 repetitions

## Discussion

To our knowledge, this is the first study focusing on the effect of exercise training on peripheral vascular function in AD and to explore the mechanisms by which exercise training induces positive peripheral vascular adaptations in this population. In the present study, we measured peripheral vascular function and plasma VEGF in individuals with AD before and after 6 months of exercise training compared with a control group. Also, we measured BF and SR during treatments and found that during EX both these parameters consistently increased. In accordance with our hypothesis, individuals with AD showed an amelioration of peripheral vascular function measured by means of PLM and FMD tests to EX. Also, a great increase in BF and SR during EX was recorded, supporting the hypothesis that EX-induced up-regulation of NO bioavailability, and plasma VEGF was likely triggered by the augmented frictional force on vessel walls during exercise training. On the contrary, individuals with AD taking part in the CTRL group did not show any adaptation of peripheral vascular functions, BF and SR.

### Evidence on the effect of exercise training on peripheral vascular functions in individuals with AD

To our knowledge, no studies have measured FMD, PLM, and VEGF in individuals with AD in response to an exercise program. However, exercise-induced peripheral vascular adaptations are well known in healthy young and old individuals. Indeed, Groot et al. (2016) investigated the effect of endurance training on PLM reporting a significant augmentation of PLM ∆peak in young individuals and two groups of elderly individuals: active and endurance trained. Also, Landers-ramos et al. (2016) tested the short-term effect of aerobic exercise training on vascular function in sedentary elderly individuals reporting a 10% amelioration in FMD after only 10 days of training. Moreover, several studies support the fact that exercise training is a strong stimulus for VEGF up-regulation (Trigiani and Hamel 2017; Uchida et al. 2015). Our results are perfectly in agreement with those of the previous studies. Indeed, individuals with AD taking part in EX showed a great amelioration in FMD%, PLM ∆peak and AUC, and a great increase in VEGF (Fig. 3).

### Physiological mechanisms involved in exercise-induced adaptations on peripheral vascular functions in individuals with AD

An amelioration of the hyperemic response during PLM is of great importance. Indeed, it has been demonstrated that hyperemic response to PLM test is strongly dependent on the amount of available NO (Green et al. 2014; Trinity et al. 2012; Groot et al. 2015). Thus, an amelioration in PLM ∆peak is likely to be related not only as an amelioration of general vascular function but also to increase NO bioavailability. In addition, an amelioration of FMD % in this population is of great interest since FMD appears to be an important, independent predictor for the development of cardiovascular diseases and an important marker for vascular disfunction (Rossman et al. 2016). Thus, our results are not only confirming the importance of exercise training in inducing positive vascular adaptations but demonstrated that AD population can greatly improve peripheral vascular function by means of exercise training.

**Fig. 4:**
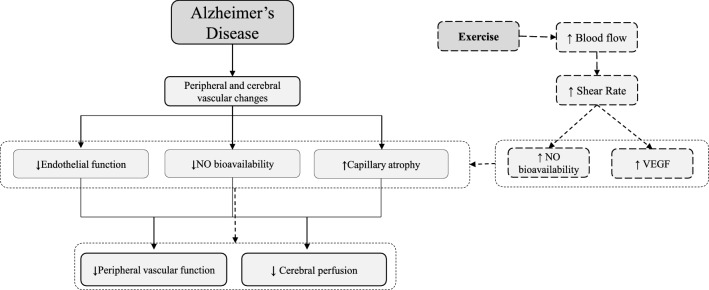
Model of the direct effect of exercise on vascular function in Alzheimer’s disease. Exercise-induced effects may act directly on the peripheral and cerebral vascular function and consequently act indirectly on AD-related symptoms and comorbidities. Indeed, during exercise, blood flow and vascular shear stress greatly increase in tissue beds with high metabolic activity, leading to the activation of endothelial NO synthase, improving NO bioavailability. In addition, in response to the increased energy demand during exercise, VEGF is upregulated, which mediates mediating in morphology, number, and diameter of capillary and arteries. This angiogenic process is associated with functional changes and improvement in organ blood flow, including cerebral blood flow (CBF), which is significantly altered in the AD process

Exercise-induced effects on peripheral vascular function might be largely explained by a variety of molecular mechanisms that provide protective environment in vascular system, and its beneficial effect can be likely extended to cerebral vasculature as well (Lange-Asschenfeldt and Kojda 2008) (Fig. 4). These positive effects might be reached by the direct action of exercise training on vascular NO metabolism and up-regulation of vascular growth factors (Lange-Asschenfeldt and Kojda 2008). Indeed, during exercise training, BF and vascular SR are elevated in tissue beds with high metabolic activity (Kutikhin et al. 2018), which leads to the activation of endothelial NO synthase, improving NO bioavailability (Fig. 4) (Trigiani and Hamel 2017). In this study, we directly measured BF and SR in individuals with AD during EX and CTRL showing that they greatly increased during both endurance and resistance exercise in EX group, but no changes were found in CTRL (Fig. 3). Thanks to its adaptive response exercise training may change the morphology of arterial vessels and increase the number and diameter of arteries in response to the up-regulation of VEGF and other growth factors, which have angiogenic and neuroprotective roles (Lange-Asschenfeldt and Kojda 2008). This angiogenic process is associated with functional changes and improvement in organ blood flow (Lange-Asschenfeldt and Kojda 2008).

### Exercise-related practical considerations

Exercise intervention included both aerobic and resistance exercises, and each exercise included in the intervention was selected for its easy execution and safety. Walking on a treadmill, cycling on a cycle-ergometer and working with the arm-cracking were all well tolerated by each participant, none of them exhibited discomfort during exercise. However, the first 4–5 exercise sessions were focused on the familiarization with the equipment and the exercises, especially with the treadmill walking which is the exercise that the most requires coordination and balance. In addition, resistance exercise was decided to include only isotonic machines, avoiding free weights. This allowed participants to be guided throughout the movement making the execution as safer as possible and correct. We should also highlight that the ratio of kinesiologists:participants was 2:5. This ratio allowed each participant to be appropriately supervised and to have the proper hint during exercise sessions. No adverse events related to the exercise intervention were recorded during the 6 months and workload was correctly increased month by month to meet the required exercise intensity. Functional adaptations were also reached as exhibited by the amelioration in 6MWT and PPT in the EX group.

However, although a significant amelioration was achieved by EX group, since the training included both aerobic and resistance exercise, we cannot state which exercise component had the most impact on vascular function.

### Clinical relevance of enhancing vascular function in AD

Because no disease-modifying strategy is available for AD, effective strategies that may improve AD comorbidities and symptoms are needed. Some reasons underscore the importance of new approaches focused on the amelioration of peripheral vascular function in AD. First, the presence of a vascular dysfunction may lower the threshold for dementia for a given AD pathology burden, meaning that the threshold for AD to become symptomatic is lowered by vascular disease (Eldholm et al. 2018). For this reason, different individuals with the same AD burden may express cognitive and behavioral symptoms depending on their vascular function. Second, enhancing peripheral vascular function can lower the risk of developing other cardiovascular diseases, which might increase AD burden (Alagiakrishnan et al. 2006; Eldholm et al. 2018). Consequently, enhancing peripheral vascular function could probably positively affect the expression of AD-related cognitive dysfunctions and symptoms.

## Conclusion

As long as a disease-modifying treatment for AD is not available, inducing a positive adaptation of other processes known to be involved in AD pathogenesis, such as peripheral vascular dysfunction, is a major focus of research and it may help in increasing the quality of life of the individuals affected by this pathology, lowering the risk of developing other comorbidities. Although AD is not reversable, this non-pharmacological approach might be included in the standard clinical practice for individuals with AD if the data from this study were to be confirmed in larger multicenter studies.

## Data Availability

Data and material are available upon request to the corresponding author.

## Abbreviations

1RM: 1-Repetition maximum
AD: Alzheimer’s disease
ADAS-Cog: Cognitive section of the Alzheimer’s disease Assessment Scale
ANOVA: Analysis of variance
AUC: Area under the curve
BF: Blood flow
CDR: Clinical Dementia Rate
CTRL: Control group
EX: Exercise training
FMD: Flow-mediated dilation
HR: Heart rate
MMSE: Mini-Mental State Examination
NO: Nitric oxide
PLM: Passive limb movement
POMA: Performance Oriented Mobility Assessment
RCT: Randomized clinical trial
RPE: Rate of perceived exertion
SR: Shear rate
VEGF: Vascular endothelial growth factor
Vmean: Mean velocity
